# Multipartite uncertainty relation with quantum memory

**DOI:** 10.1038/s41598-021-93044-8

**Published:** 2021-07-02

**Authors:** Saeed Haddadi, Mohammad Reza Pourkarimi, Soroush Haseli

**Affiliations:** 1 Faculty of Physics, Semnan University, P.O.Box 35195-363, Semnan, Iran; 2 Saeed’s Quantum Information Group, P.O.Box 19395-0560, Tehran, Iran; 3 Department of Physics, Salman Farsi University of Kazerun, Kazerun, Iran; 4 Faculty of Physics, Urmia University of Technology, Urmia, Iran

**Keywords:** Information theory and computation, Quantum physics

## Abstract

We present a new quantum-memory-assisted entropic uncertainty relation for multipartite systems which shows the uncertainty principle of quantum mechanics. Notably, our results recover some well-known entropic uncertainty relations for two arbitrary incompatible observables that demonstrate the uncertainties about the results of two measurements. This uncertainty relation might play a critical role in the foundations of quantum theory.

The uncertainty principle is a special feature of quantum mechanics. Historically, the first uncertainty relation formulated by Heisenberg^1^ who showed that one cannot predict the results with arbitrary precision for two incompatible observables concurrently, such as measurements of position *x* and momentum *p* of a particle^2^. Following, Robertson^3^ proposed an inequality in terms of a standard deviation in respect to a pair of incompatible observables for the systemic state. Nevertheless, Robertson’s uncertainty bound is state-dependent and so will be trivial for some states. In order to eliminate this defect, Deutsch^4^ proposed to utilise the Shannon entropy as a proper criterion of uncertainty and introduced the so-called entropic uncertainty relation (EUR). Moreover, Kraus^5^ improved Deutsch’s uncertainty bound, and later Maassen and Uffink^6^ proved it as

1 $$\begin{aligned} H(Q)+H(R)\ge -\log _{2}(c), \end{aligned}$$

where $H(Q)=-\sum _{i}q_{i}\log _{2} q_{i}$ and $H(R)=-\sum _{j}r_{j}\log _{2} r_{j}$ are the Shannon entropy of the probabilities of measurement results of the incompatible observables *Q* and *R*, respectively. The complementarity $c=\max _{i,j}\{|\langle q_{i}|r_{j}\rangle |^{2}\}$ is the maximal overlap of *Q* and *R* with $|q_{i}\rangle$ and $|r_{j}\rangle$ being the eigenstates of *Q* and *R*, respectively. Compared with Robertson’s uncertainty bound, the bound of EUR (1) depends only on the complementarity of the two observables, avoiding the deficiency of state dependence. Noteworthily, a recent study has shown that the variance-based and entropy-like uncertainty relations are mutually equivalent^7^.

After that, a striking result of the uncertainty principle is to study the effect of a quantum memory that is achievable with current technology. Berta et al.^8^ revealed that the entropic uncertainty can be decreased with the help of memory particle which might be correlated with the measured system. They demonstrated a new uncertainty relation, which can be called quantum-memory-assisted entropic uncertainty relation (QMA-EUR). This relation can be described by the interesting uncertainty game between two legitimate players, Alice and Bob. At the beginning of the game, Bob has a pair of correlated particles, *A* and *B*, and sends particle *A* to Alice, with *B* as a memory particle. In the next step, she carries out a measurement on her quantum system by choosing one of the observables *Q* and *R*. Afterwards, she announces to Bob her choice of the measurement. Finally, Bob’s task is to predict Alice’s measurement outcome. During this game, the uncertainty via von-Neumann entropies can be written as

2 $$\begin{aligned} S(Q|B)+S(R|B) \ge -\log _{2} (c) + S(A|B), \end{aligned}$$

where $S(A|B)=S(\rho _{AB})-S(\rho _{B})$ is the conditional von-Neumann entropy of $\rho _{AB}$ with $\rho _{B}=tr_{A}(\rho _{AB})$ and $S(\rho )=-tr(\rho \log _{2}\rho )$ is the von-Neumann entropy. Also, $S({\mathcal {O}}|B)=S\left( \rho _{\mathcal {O} B}\right) -S\left( \rho _{B}\right)$ with ${\mathcal {O}} \in \{Q, R\}$ is the conditional von-Neumann entropy of the post-measurement state after the quantum system *A* is measured, $\rho _{Q B}=\sum _{i}(\left| q_{i}\right\rangle _{A}\left\langle q_{i}\right| \otimes {\mathbb {I}}_{B}) \rho _{A B}(\left| q_{i}\right\rangle _{A}\left\langle q_{i}\right| \otimes {\mathbb {I}}_{B}),$ likewise for $\rho _{R B}$, and ${\mathbb {I}}_{B}$ being an identity operator in Hilbert space of *B*.

In the literature, substantial efforts have been made to improve Berta et al.’s bound^9–17^. To be explicit, Pati et al.^9^ improved Berta et al.’s bound by a term added to the right-hand side of inequality (2). Indeed, Pati et al.’s bound is tighter than Berta et al.’s bound if the quantum discord is larger than the classical correlation. Then, Pramanik et al.^10^ obtained a new entropic uncertainty relation based on fine graining, which led to an ultimate limit on the accuracy achievable in measurements made on two incompatible observables in the presence of quantum memory. Moreover, Coles and Piani^11^ reported a strong bound by considering the second largest value of the overlap. Zhang et al.^12^ presented tighter bounds on both entropic uncertainty and information exclusion relations for multiple measurements in the presence of quantum memory. In Ref.^13^, Pramanik et al. derived a new form of the uncertainty relation through extractable classical information. In 2016, Xiao et al.^14^ tightened Berta et al.’s bound based on Coles and Piani’s remarkable bound. Later, Chen et al.^15^ improved the lower bounds for the entropic uncertainty relations via polynomial functions. Besides, Huang et al.^16^ presented a Holevo bound for QMA-EUR, where the difference between the entropic uncertainties and the new bound is always a fixed value. More recently, Li and Qiao^17^ proposed a method to decrease the local uncertainty. In this remarkable study a new kind of uncertainty relation based on conditional majorization^18–20^ has been formulated, which can be calculated for any number of observables. According to this new class of uncertainty relation and in the presence of quantum memory, one can get lower bounds in comparison to the conditional entropic uncertainty relation. On the other hand, continuing progress has been done by some groups from an experimental viewpoint^21–31^. Furthermore, a promising effort was made by Adabi et al.^32^ to tighten the Berta et al.’s bound, which is expressed as

3 $$\begin{aligned} {S(Q|B)+S(R|B) \ge -\log _{2} (c) + S(A|B)+\max \{0, \delta \},} \end{aligned}$$

where $\delta =I(A:B)-[I(Q:B)+I(R:B)]$, $I(A:B)=S(\rho _{A})+S(\rho _{B})-S(\rho _{AB})$ is mutual information between Alice and Bob, and $I({\mathcal {O}}:B)=S\left( \rho _{B}\right) -\sum _{i} p_{i} S\left( \rho _{B|i}\right)$ is Holevo quantity. Note that, $\rho _{B|i}=tr_{A}(\Pi _{i}^{A}\rho _{AB}\Pi _i^{A})/p_i$ is the post-measurement state of Bob after measuring of observable ${\mathcal {O}}$ by Alice and $p_i=tr_{AB}(\Pi _{i}^{A}\rho _{AB}\Pi _i^{A})$ is the probability of *i*th outcome.

Interestingly, the bipartite QMA-EUR has been the topic of many works in recent years^33–48^ (see Refs.^49^ and^50^ for detailed reviews on bipartite QMA-EUR). However, Renes and Boileau^51^ showed that the bipartite QMA-EUR could be generalized to the tripartite case where two particles *B* and *C* are considered as the quantum memories. It is shown that the tripartite QMA-EUR can be written as

4 $$\begin{aligned} S(Q|B)+S(R|C) \ge -\log _{2} (c). \end{aligned}$$

In this scenario, Alice, Bob, and Charlie share a tripartite quantum state $\rho _{ABC}$ and then Alice carries out one of two observables (*Q* and *R*) on her system. Briefly, if Alice measures *Q*, then Bob’s task is to minimize his uncertainty about *Q* and whenever she measures *R*, then Charlie’s task is to minimize his uncertainty about *R*. Most recently, Ming et al.^52^ improved the tripartite uncertainty bound of the inequality (4) as

5 $$\begin{aligned} S(Q|B)+S(R|C) \ge -\log _{2} (c)+\max \{0, \Delta \}, \end{aligned}$$

where

6 $$\begin{aligned} \Delta =&-\log _{2} (c)+2 S(A)-[I(A:B)+I(A:C)]+[I(Q:B)+I(R:C)]-H(Q)-H(R). \end{aligned}$$

Subsequently, Dolatkhah et al.^53^ introduced a new lower bound for tripartite QMA-EUR that is tighter than Ming et al.’s bound. The new inequality can be derived by

7 $$\begin{aligned} S(Q|B)+S(R|C) \ge&-\log _{2} (c)+\frac{S(A|B)+S(A|C)}{2}+\max \{0, \delta ^{\prime }\}, \end{aligned}$$

with

8 $$\begin{aligned} \delta ^{\prime }= \frac{I(A:B)+I(A:C)}{2}-[I(Q:B)+I(R:C)]. \end{aligned}$$

Within the above, it is obvious that the studies only have been focused on the case of two measurements (*Q* and *R*). However, many attempts have been made to generalize the entropic uncertainty relations to more than two observables^54–63^. Here, one can refer to the result of Liu et al.^55^ that they considered Maassen and Uffink bound for multi-observable ($>2$) with the state of the measured system $A\equiv \rho _{A}$ which is generally a mixed state, viz

9 $$\begin{aligned} \sum _{m=1}^{N} H({\mathcal {O}}_{m}) \ge -\log _2 (b) + (N-1)S(A), \end{aligned}$$

in which

10 $$\begin{aligned} b=\max _{i_{N}}\left\{ \sum _{i_{2} \sim i_{N-1}} \max _{i_{1}}\left[ c(u_{i_{1}}^{1}, u_{i_{2}}^{2})\right] \prod _{m=2}^{N-1} c(u_{i_{m}}^{m}, u_{i_{m+1}}^{m+1})\right\} , \end{aligned}$$

where $c(u_{i_{1}}^{1}, u_{i_{2}}^{2})=|\langle u_{i_{1}}^{1} \mid u_{i_{2}}^{2}\rangle |^{2}$ and $c(u_{i_{m}}^{m}, u_{i_{m+1}}^{m+1})=|\langle u_{i_{m}}^{m} \mid u_{i_{m+1}}^{m+1}\rangle |^{2}$ with $\left| u_{i_{m}}^{m}\right\rangle$ which is the *i*th eigenstate of ${\mathcal {O}}_{m}$. Furthermore, this inequality in the presence of quantum memory *B* is converted to^55^

11 $$\begin{aligned} \sum _{m=1}^{N} H({\mathcal {O}}_{m}|B) \ge -\log _2 (b) + (N-1)S(A|B). \end{aligned}$$

Until now, the inequalities revealed only for bipartite, tripartite, and multi-measurement cases, while the case of multipartite systems remains unstudied. In this paper, we will present a novel entropic uncertainty with quantum memory for multipartite systems where the memory is split into several parts. Herein, we highlight this relation for its key role in quantum theory and potential wide applications, as well as expect that this uncertainty relation can be demonstrated in various physical systems.

## Generalization of QMA-EUR

The following theorem reveals how to obtain the QMA-EUR for multipartite systems.

### Theorem 1

By considering the measured system state $A\equiv \rho _{A}$ that is generally mixed, the following multipartite uncertainty relation holds for any state $\rho _{Am}$ ($m=1,2,...,N$)

12 $$\begin{aligned} \sum _{m=1}^{N} S({\mathcal {O}}_{m}|P_{m}) \ge&-\log _2 (b) + \frac{N-1}{N} \sum _{m=1}^{N} S(A|P_{m})+\max \{0,\kappa \}, \end{aligned}$$

where

13 $$\begin{aligned} \kappa =\frac{N-1}{N} \sum _{m=1}^{N} I(A:P_{m})-\sum _{m=1}^{N} I({\mathcal {O}}_{m}:P_{m}), \end{aligned}$$

with ${\mathcal {O}}_{m}$ and $P_{m}$ which are the different incompatible observables and the memory particles for *m*th measurement, respectively. In this scenario, Alice and the others share a multipartite quantum state $\rho _{Am}$ and then Alice carries out one of the observables (${\mathcal {O}}_{m}, m=1,2,...,N$) on her system. If Alice measures ${\mathcal {O}}_{1}$, then $P_{1}$ has the task to minimize his uncertainty about ${\mathcal {O}}_{1}$. If she measures ${\mathcal {O}}_{2}$, then $P_{2}$ has the task to minimize his uncertainty about ${\mathcal {O}}_{2}$. Generally, whenever Alice measures ${\mathcal {O}}_{m}$, then $P_{m}$ has the task to minimize his uncertainty about ${\mathcal {O}}_{m}$.

### Proof

Regarding $S({\mathcal {O}}|P)=H({\mathcal {O}})-I({\mathcal {O}}:P)$, the left-hand side of inequality (12) can be rewritten as

14 $$\begin{aligned} \sum _{m=1}^{N} S({\mathcal {O}}_{m}|P_{m})=&\sum _{m=1}^{N} H({\mathcal {O}}_{m})- \sum _{m=1}^{N} I({\mathcal {O}}_{m}:P_{m})\nonumber \\ \ge&-\log _{2}(b)+(N-1) S(A)- \sum _{m=1}^{N} I({\mathcal {O}}_{m}:P_{m})\nonumber \\ =&-\log _{2}(b)+(N-1) [S(A|P_{1})+I(A:P_{1})]- \sum _{m=1}^{N} I({\mathcal {O}}_{m}:P_{m}), \end{aligned}$$

where the inequality follows from Liu et al.’s result for *N* measurements (9) and the last equation comes from the identity $S(A)=S(A|P_{1})+I(A:P_{1})$. By using $S(A)=S(A|P_{2})+I(A:P_{2})$ we have

15 $$\begin{aligned} \sum _{m=1}^{N} S({\mathcal {O}}_{m}|P_{m})\ge&-\log _{2}(b)+(N-1) [S(A|P_{2})+I(A:P_{2})]- \sum _{m=1}^{N} I({\mathcal {O}}_{m}:P_{m}). \end{aligned}$$

In general, for $m=1,2,\ldots ,N$ by substituting the identity $S(A)=S(A|P_{m})+I(A:P_{m})$ into (14), one arrives at

16 $$\begin{aligned} \sum _{m=1}^{N} S({\mathcal {O}}_{m}|P_{m})\ge&-\log _{2}(b)+\frac{N-1}{N} \sum _{m=1}^{N} S(A|P_{m})+\frac{N-1}{N} \sum _{m=1}^{N} I(A:P_{m})- \sum _{m=1}^{N} I({\mathcal {O}}_{m}:P_{m}), \end{aligned}$$

which can be rewritten as the desired outcome (12). $\square$

### Corollary 1

If the prepared state is a bipartite state, our uncertainty relation will recover Adabi et al.’s result.

### Proof

For any bipartite state, we consider two observables (${\mathcal {O}}_{1}=Q$ and ${\mathcal {O}}_{2}=R$) and $P_{1}=P_{2}=B$. Therefore, from Eq. (12) we obtain the Eq. (3) with the complementarity $b=c$ and $\kappa =\delta$.$\square$

### Corollary 2

If the prepared state is a tripartite state, our uncertainty relation recover Dolatkhah et al.’s result.

### Proof

For any tripartite state, we consider two observables (${\mathcal {O}}_{1}=Q$ and ${\mathcal {O}}_{2}=R$) and $P_{1}=B$ and $P_{2}=C$. So, we restore the Eq. (7) with the complementarity $b=c$ and $\kappa =\delta ^{\prime }$. $\square$

## Example: four-partite QMA-EUR

Let ${\mathcal {O}}_{1}$, ${\mathcal {O}}_{2}$, and ${\mathcal {O}}_{3}$ be three incompatible observables for a four-partite system $\rho _{ABCD}$ which is generally a mixed state. The following four-partite uncertainty relation holds (with $P_{1}=B$, $P_{2}=C$, and $P_{3}=D$)

17 $$\begin{aligned} S({\mathcal {O}}_{1}|B)+S({\mathcal {O}}_{2}|C)+S({\mathcal {O}}_{3}|D) \ge&-\log _2 (b^{\prime })+ \frac{2}{3} [S(A|B)+S(A|C)+S(A|D)]+\max \{0,\kappa ^{\prime }\}, \end{aligned}$$

where

18 $$\begin{aligned} \kappa ^{\prime }=&\frac{2}{3} [I(A:B)+I(A:C)+I(A:D)] - I({\mathcal {O}}_{1}:B)- I({\mathcal {O}}_{2}:C)- I({\mathcal {O}}_{3}:D), \end{aligned}$$

and

19 $$\begin{aligned} b^{\prime }=\max _{k}\left\{ \sum _{j} \max _{i}\left[ \left| \left\langle u_{i}^{1} \mid u_{j}^{2}\right\rangle \right| ^{2}\right] |\langle u_{j}^{2} \mid u_{k}^{3}\rangle |^{2}\right\} , \end{aligned}$$

with $|u_{i}^{1}\rangle$, $|u_{j}^{2}\rangle$ and $|u_{k}^{3}\rangle$ being the eigenstates of the three observables ${\mathcal {O}}_{1}$, ${\mathcal {O}}_{2}$ and ${\mathcal {O}}_{3}$, respectively.

It is worth noting that the memory has been split into three parts. In this situation, after preparing a four-partite state $\rho _{ABCD}$, particle *A* is sent to Alice, *B* to Bob, *C* to Charlie, and *D* to David. Now, Alice carries out one of the three observables (${\mathcal {O}}_{m}, m=1,2,3$) on her system. Then, Alice informs Bob, Charlie, and David of her measurement choice. If Alice measures ${\mathcal {O}}_{1}$, then Bob’s task is to minimize his uncertainty about ${\mathcal {O}}_{1}$. If she measures ${\mathcal {O}}_{2}$, then Charlie’s task is to minimize his uncertainty about ${\mathcal {O}}_{2}$. Finally, if she measures ${\mathcal {O}}_{3}$, then David’s task is to minimize his uncertainty about ${\mathcal {O}}_{3}$.

## Conclusion

In summary, we have presented a generalized uncertainty relation with quantum memory for multipartite systems and obtained a new QMA-EUR for four-partite quantum systems. This generalized entropic uncertainty depends on the conditional von-Neumann entropies, Holevo quantities, and the mutual information. We expect that the inequality will bring on more potential applications in quantum information and communication, e.g., entanglement detection^64^, multipartite entanglement-structure detection^65^, witnessing multipartite entanglement^66^, detection of genuine multipartite entanglement in multipartite systems^67^, exploring the efficient multipartite entanglement criteria^68–70^, analyzing the monogamy and polygamy relations of multipartite quantum states^71,72^, and so on. It means that our multipartite uncertainty relation will have significant applications in entanglement detection and precision measurements. In a forthcoming paper, one may motivate to extend the results to get an uncertainty relation for multipartite systems based on conditional majorization in comparison to recent study^17^.
